# Gestational diabetes mellitus: Case definition & guidelines for data collection, analysis, and presentation of immunization safety data

**DOI:** 10.1016/j.vaccine.2017.01.043

**Published:** 2017-12-04

**Authors:** Alisa Kachikis, Linda O. Eckert, Christie Walker, Eugene Oteng-Ntim, Rama Guggilla, Manish Gupta, Manasi Patwardhan, Ronald Mataya, Tamala Mallett Moore, Ana Maria Alguacil-Ramos, Cheryl Keech, Michael Gravett, Helen Murphy, Sonali Kochhar, Nancy Chescheir, The Brighton Collaboration Gestational Diabetes Mellitus Working Group

**Affiliations:** a University of Washington, Seattle, WA, USA; b London School of Hygiene and Tropical Medicine, UK; c King’s College London, UK; d George Institute for Global Health, India; e Barts Health NHS Trust, London, UK; f Wayne State University, USA; g Loma Linda University, USA; h University of Malawi College of Medicine, Malawi; i Sanofi Pasteur, USA; j Dirección General de Salud Pública, Conselleria de Sanidad Universal y Salud Pública, Spain; k Fundación para el Fomento de la Investigación Sanitaria y Biomédica (FISABIO), Spain; l PPD, USA; m Global Alliance to Prevent Prematurity and Stillbirth, An Initiative of Seattle Children’s Hospital, USA; n University of East Anglia/Cambridge University Hospitals NHS Foundation Trust, UK; o Global Healthcare Consulting, Delhi, India; p University of North Carolina, Chapel Hill, USA; q Erasmus University Medical Center, Rotterdam, The Netherlands

## 1 Preamble

### 1.1 Need for developing case definitions and guidelines for data collection, analysis, and presentation for gestational diabetes mellitus as an adverse event following immunization

Gestational diabetes mellitus (GDM) is a common condition in pregnancy that can result in significant morbidity and mortality to both mother and fetus. According to the International Diabetes Federation (IDF), about 16.8% of live-births are born to women with hyperglycemia in pregnancy [1]. Approximately 16% of these women will have pre-existing diabetes mellitus, diagnosed prior to pregnancy or during the first trimester of pregnancy. The remainder will have GDM. The incidence of GDM follows the incidence of insulin-resistance and type 2 diabetes mellitus (T2DM) in a given country’s population [2]. The prevalence of GDM can range anywhere from 1% to 15% depending on screening methods used, risk factors and ethnicity [3]. The Global Burden of Disease Project and IDF estimate that the rates of T2DM, including those of reproductive-age women, will continue to rise annually especially in low- and middle-income countries (LMICs) due to increasing risk factors such as obesity and sedentary lifestyle [4].

The pathophysiology for GDM centers around the inability of a pregnant woman to develop an adequate insulin response to a glucose load to maintain her blood sugar in a normal range. This is due to decreasing insulin sensitivity as the pregnancy progresses. Risk factors for GDM include family history of diabetes, GDM in prior pregnancy, ethnicity and obesity. However it has been found that screening based on these factors will miss approximately 50% of women with GDM [5]. GDM places mothers at increased risk for gestational hypertension, pre-eclampsia and cesarean section during pregnancy [6]. In addition, women with a history of GDM are at higher risk for developing T2DM in the future [7]. Fetal complications of pregnancies with GDM include increased risk of macrosomia, operative delivery, shoulder dystocia, birth trauma and neonatal hypoglycemia and hyperbilirubinemia. The Hyperglycemia and Adverse Pregnancy Outcome (HAPO) study demonstrated a continuous association between maternal glucose levels and increased birth weight, cesarean section deliveries and neonatal hyperinsulinemia [8]. In addition, in utero exposure to maternal hyperglycemia may predispose to obesity and insulin resistance later in life [9], [10]. Given the risk for significant maternal and fetal morbidity and mortality in pregnancies complicated by GDM, strict glycemic control during pregnancy is recommended [11], [12] it is also important to be cognizant of medications that may cause transient hyperglycemia or that exacerbate hyperglycemia in mothers with GDM, such as beta-adrenergic agents and corticosteroids that are often administered to women with threatened preterm labor [13].

The association between maternal immunization and GDM, whether the development or exacerbation of the disease, or even the mitigation of disease, has not been well studied and is unknown. Multiple large prospective and retrospective vaccination studies have included GDM as a potential adverse outcome, often relying on ICD-9 or ICD-10 codes for diagnosis. While these studies did not find an increased incidence of GDM after maternal immunization, they had multiple confounders [14], [15], [16], [17], [18], [19], [20], [21], [22]. One of these confounders is the specific timing of vaccinations in pregnancy in relation to the diagnosis of GDM. Vaccinations are predominantly administered in the first and third trimesters, and GDM testing and diagnosis occurs in the second and third trimesters. This inevitably leads to the diagnosis of GDM after vaccine administration has occurred or concurrently. This timing makes determining the actual effect of vaccines on glucose tolerance difficult. Other medications, such as corticosteroids and beta-mimetics, are known to alter glucose tolerance, although only transiently [13]. Ideally, studies that examine the effect of vaccines on glucose tolerance would include a time period close to the vaccination administration, perhaps 0–14 days. Since this definition is not currently in use the studies included in this review have not limited the time between vaccination and diagnosis of GDM. Another confounder to the prior studies is that the true incidence of GDM is not known and ranges widely based on the population examined and the diagnostic criteria used [23]. If the baseline incidence in the study population is not known then determining the change in incidence after vaccination is not feasible. For this review, we have focused on the development of GDM as a possible adverse event following vaccination. We have excluded treatment of GDM, as well as maternal, fetal and neonatal complications attributed to GDM as these poor outcomes were likely due to GDM and other co-morbidities, not directly to the vaccine.

There is wide variation for the diagnostic criteria for GDM globally depending on country, consensus statements and resources available. Recommendations for GDM screening in pregnancy, usually between 24 and 28 weeks gestational age, are increasingly becoming universal, however, are often based on risk factors in resource-limited settings. While the gold standard for diagnosis of GDM is an oral glucose tolerance test, the blood glucose cut-offs often vary between and within countries, and sampling methodology can range from laboratory results based on venous serum samples to plasma samples using calibrated handheld glucometers [24]. In resource-limited settings, alternative methods of diagnosis have been proposed including fasting glucose levels, glucosuria or diagnosis based on other risk factors.

There is hence no uniformly accepted definition of Gestational Diabetes Mellitus. This is a missed opportunity, as data comparability across trials or surveillance systems would facilitate data interpretation and promote the scientific understanding of GDM in general, and for our purposes, any possible relationship of maternal vaccination with the development of GDM.

### 1.2 Methods for the development of the case definition and guidelines for data collection, analysis, and presentation for gestational diabetes mellitus as an adverse events following immunization

Following the process described on the Brighton Collaboration Website http://www.brightoncollaboration.org/internet/en/index/process.html, the Brighton Collaboration Gestational Diabetes Working Group was formed in 2016 and included members from clinical, academic, public health, and industry backgrounds. The composition of the working and reference group as well as results of the web-based survey completed by the reference group with subsequent discussions in the working group can be viewed at: http://www.brightoncollaboration.org/internet/en/index/working_groups.html.

To guide the decision-making for the case definition and guidelines, a literature search was performed using MEDLINE and Embase databases. Due to the extensive and diverse topic of gestational diabetes mellitus, the search was limited to systematic reviews conducted in the prior five years. The search term for Pubmed is shown below and was modified for Embase search terminology:

**((“pregnancy induced diabetes” AND diagnosis)** Filters: **published in the last 5 years; Humans; English)**
**OR**
**(gestational diabetes/diagnosis OR (Diabetes mellitus AND diagnosis AND pregnancy) OR “diabetes in pregnancy” OR Glucose intolerance of pregnancy OR hyperglycemia in pregnancy OR hyperglucosuria)** Filters: **Systematic Reviews; published in the last 5 years; Humans; English)**

A separate search was done to identify any studies or reports associating gestational diabetes mellitus with immunizations and vaccinations, using MEDLINE, Embase, the Cochrane Database of Systematic Reviews, Clinical Key medical reference books, and the Centers for Disease Control and Prevention (CDC) and National Institutes for Health (NIH) websites. The following search string was used:

((**maternal** NEXT/1 (**vaccin^∗^** OR **immuniz^∗^** OR **immunis^∗^**)) OR (((**'vaccine'**/exp/mj OR **'immunization'**/exp/mj OR **vaccin^∗^**:ti OR **immuniz^∗^**:ti OR **immunis^∗^**:ti OR **revaccin^∗^**:ti OR **postvaccin^∗^**:ti OR **reimmuni^∗^**:ti OR **postimmuni^∗^**) AND (**'pregnancy'**/exp/mj OR **'child bearing'**:ti OR **'childbearing'**:ti OR **'gestation'**:ti OR **'gravidity'**:ti OR ((**labor** OR **labor**) NEXT/1 **presentation**):ti OR**pregnan^∗^**:ti OR **'pregnant woman'**/exp/mj OR **'expectant mother'**/exp/mj OR (**expectant** NEXT/1 **mother^∗^**) AND (**'pregnancy diabetes mellitus'**/exp AND **diabetes** NEAR/2 (**gestational** OR **pregnancy**))

Two committee members reviewed the literature search results for duplications and appropriateness to the topic, retaining 111 of 209 included documents. Of the search for gestational diabetes related to vaccinations, 11 of 14 were retained after review. Differences in literature review were adjudicated by a third committee member.

**Fig. 1:**
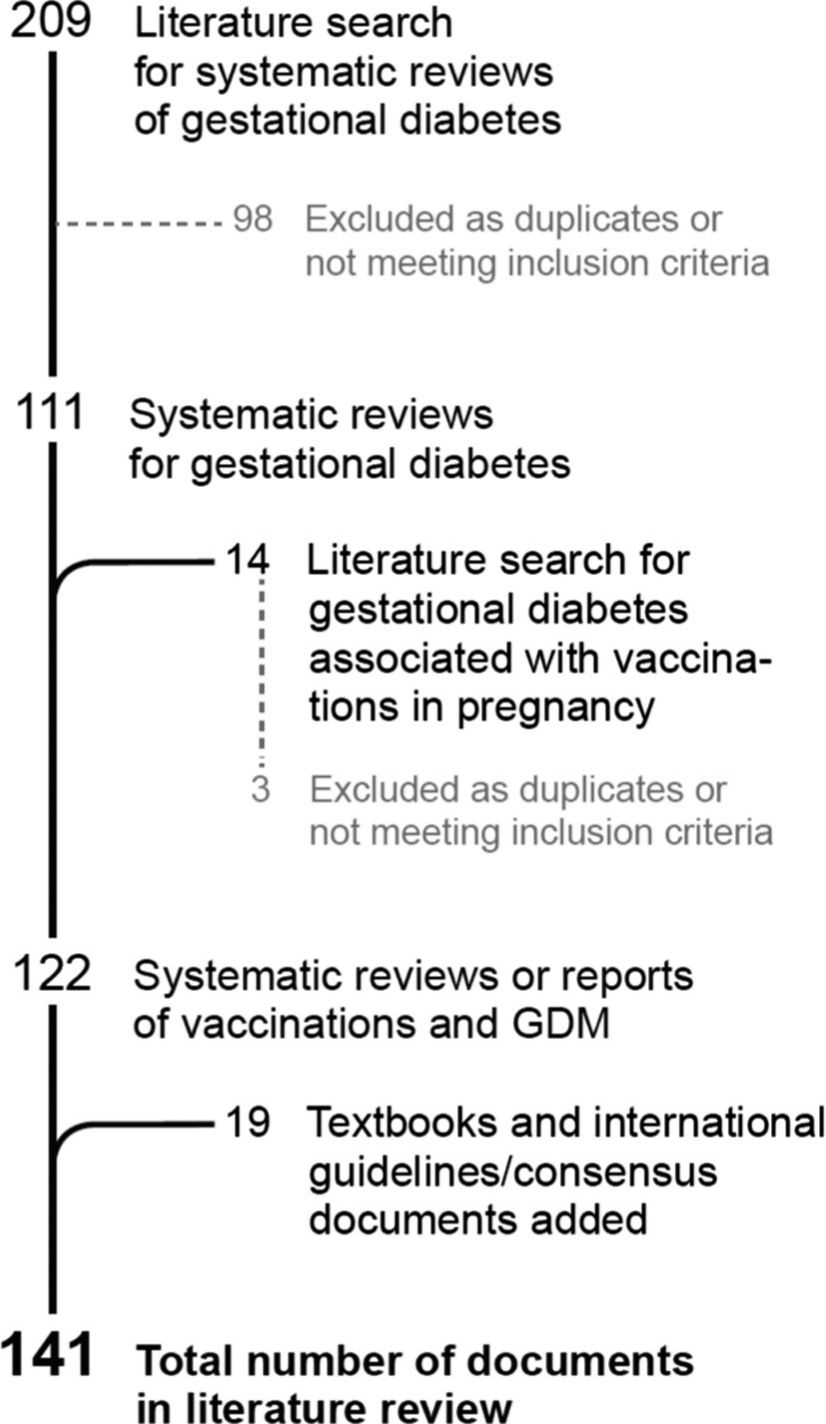
Flow diagram describing pathway for source identification.

In addition, we identified latest edition Obstetrics and Gynecology text books in common usage in North America, Europe and Africa and reviewed four of these for definitions of GDM. Fifteen national endocrine and obstetrics and gynecology guidelines were reviewed. A flow diagram of identified sources is shown in Fig. 1.

Each member of the Gestational Diabetes work group was assigned approximately eight of the 141 articles to review for identification of a working definition of gestational diabetes and the preferred method of diagnosing it or for describing any association of gestational diabetes as a complication of vaccination.

Findings of the literature search included varying definitions of gestational diabetes mellitus in the literature as well as different diagnostic criteria described in the systematic reviews and research studies. In the majority of cases, oral glucose tolerance tests were used to diagnose gestational diabetes with venous blood draws. The specific glucose tolerance test as well as the glucose level cut-offs vary between reviews and consensus guidelines. An inventory spreadsheet of definitions and diagnostic criteria of the 141 pieces of literature as well as a summary page comparing the most common guidelines for the definition of gestational diabetes was made available to working group members. The full reference list of documents, consensus guidelines and textbooks are available upon request. Please contact the corresponding author for further information.

### 1.3 Rationale for selected decisions about the case definition of gestational diabetes mellitus as an adverse event following immunization

#### 1.3.1 The term gestational diabetes mellitus

– Different terminology

Alternate terminology for GDM includes “pregnancy-induced hyperglycemia.” Diabetes in pregnancy is frequently used to describe pregestational diabetes or is used as an umbrella term for pregestational diabetes AND GDM.

#### 1.3.2 Related term(s) of gestational diabetes mellitus

Pregestational diabetes mellitus (DM): Pregestational DM is the diagnosis of diabetes mellitus prior to pregnancy. Two subtypes are frequently described:

– Type 1 DM: Type 1 DM typically has an onset early in life usually due to an autoimmune process. It necessitates insulin therapy. It is commonly considered to result from insufficient production of insulin in the pancreas.
– Type 2 DM: Type 2 DM is the more common form of pregestational DM. It is characterized by insulin resistance or relative insulin deficiency and can be treated by dietary and lifestyle modifications, oral agents and/or insulin therapy.

#### 1.3.3 Formulating a case definition that reflects diagnostic certainty: weighing specificity versus sensitivity

It needs to be emphasized that the grading of definition levels is entirely about diagnostic certainty, not clinical severity of an event. Thus, a clinically very severe event may appropriately be classified as Level Two or Three rather than Level One if it could reasonably be of non-GDM etiology. Detailed information about the severity of the event should additionally always be recorded, as specified by the data collection guidelines.

The number of symptoms and/or signs that will be documented for each case may vary considerably. The case definition has been formulated such that the Level 1 definition is highly specific for the condition. As maximum specificity normally implies a loss of sensitivity, two additional diagnostic levels have been included in the definition, offering a stepwise increase of sensitivity from Level One down to Level Three, while retaining an acceptable level of specificity at all levels. In this way it is hoped that all possible cases of GDM can be captured.

#### 1.3.4 Rationale for individual criteria or decision made related to the case definition

##### 1.3.4.1 Laboratory findings

Laboratory findings are crucial to the diagnosis of GDM. Please see laboratory criteria listed below (Section 2).

#### 1.3.5 Timing post immunization

Timing criteria for considering GDM as a possible adverse event from vaccination are important. We considered only vaccinations given during pregnancy, not prior to pregnancy. GDM is usually diagnosed in the second or third trimester of pregnancy, related to increasing pregnancy-related insulin resistance. Some medications used in pregnancy, such as corticosteroids and beta-mimetics, which can result in transient hyperglycemia which can last from hours to several days. Given the paucity of identified information about GDM associated with vaccination, we are unable to provide an evidence-based estimate of time interval for the possible development of GDM following maternal immunization. Future studies on time intervals between maternal immunizations and GDM are needed (please see Section 3.2).

Timed criteria should be used, since development of hyperglycemia in pregnancy can occur at any time in the second and third trimesters of pregnancy and vaccinations are usually administered in the 1st and 3rd trimesters of pregnancy.

We postulate that a definition designed to be a suitable tool for testing causal relationships requires ascertainment of the outcome (e.g. GDM) independent from the exposure (e.g. immunizations). Therefore, to avoid selection bias, a restrictive time interval from immunization to onset of GDM should not be an integral part of such a definition. Instead, where feasible, details of this interval should be assessed and reported as described in the data collection guidelines.

Further, GDM usually occurs outside the controlled setting of a clinical trial or hospital. In some settings it may be impossible to obtain a clear timeline of the event, particularly in less developed or rural settings. In order to avoid selecting against such cases, the Brighton Collaboration case definition avoids setting arbitrary time frames.

#### 1.3.6 Differentiation from other (similar/associated) disorders

– Pregestational DM: It can be difficult to distinguish pregestational DM from GDM, especially if there is late entry to prenatal care.
– Elements to differentiate pregestational DM from GDM include timing and trimester of diagnosis and severity of hyperglycemia as well as postnatal testing results. Further details are available in Section 2. Please see below.

### 1.4 Guidelines for data collection, analysis and presentation

As mentioned in the overview paper, the case definition is accompanied by guidelines that are structured according to the steps of conducting a clinical trial, i.e. data collection, analysis and presentation. Neither case definition nor guidelines are intended to guide or establish criteria for management of ill infants, children, or adults. Both were developed to improve data comparability.

### 1.5 Periodic review

Similar to all Brighton Collaboration case definitions and guidelines, review of the definition with its guidelines is planned on a regular basis (i.e. every three to five years) or more often if needed.

## 2 Case definition of gestational diabetes mellitus^3^

### 2.1 For all levels of diagnostic certainty

Gestational diabetes mellitus (GDM) is a clinical syndrome characterized by

The absence of pre-gestational diabetes diagnosis defined by

• Previous diagnosis of diabetes while not pregnant
OR
• First trimester hemoglobin A1c level of ⩾ 6.5% (47.5 mmol/mol)
OR
• First trimester fasting blood glucose 126 mg/dL/⩾7 mmol/L

AND

Identification of sustained hyperglycemia during pregnancy not due to other known causes (i.e. corticosteroids, beta-mimetics, etc.)

### 2.2 Level 1 of diagnostic certainty

Absence of pregestational diabetes mellitus diagnosis in the first trimester as defined above with level 1–2 certainty for gestational age using GAIA definition for gestational age (please see Appendix A)

AND

Diagnosis of gestational diabetes based on a positive internationally recognized oral glucose tolerance test (see below “major criteria”) using venous blood sample/samples.

### 2.3 Level 2 of diagnostic certainty

Absence of pregestational diabetes mellitus diagnosis in the first trimester as defined above with level 1–2 certainty for gestational age using GAIA definition for gestational age (please see Appendix A)

AND

Diagnosis of gestational diabetes based on positive internationally recognized oral glucose tolerance test (see below “major criteria”) using capillary blood sample/samples.

### 2.4 Level 3 of diagnostic certainty

Absence of pregestational diabetes mellitus diagnosis in the first trimester as defined above with at least level 3 certainty for gestational age using GAIA definition for gestational age (please see Appendix A)

AND

Diagnosis of gestational diabetes based on positive internationally recognized oral glucose tolerance test (see below “major criteria”) using venous blood or capillary blood sample/samples

OR

Diagnosis of gestational diabetes based on fasting plasma glucose of 5.1–6.9 mmol/l (92–125 mg/dL) using venous or capillary blood samples.

Glucometers should be calibrated according to local standards/research protocols.

All participants in maternal immunization trials should have at minimum a fasting venous blood or capillary glucose sample prior to vaccination.

### 2.5 Insufficient evidence for diagnosis of gestational diabetes mellitus

Blood glucose cannot be measured

OR

Elevated postprandial blood glucose level without confirmatory fasting venous blood or capillary glucose level

OR

Use of Hemoglobin A1c alone for the diagnosis of GDM without a diagnostic oral glucose tolerance test (OGTT) or elevated fasting plasma glucose level

OR

Clinical and laboratory findings such as glucosuria, fundal height greater than dates, obesity, prior history of GDM or family history for the diagnosis of gestational diabetes mellitus without a diagnostic test.

### 2.6 Major and minor criteria used in the case definition of gestational diabetes mellitus

**Table:** 

Major criteria	
Endocrine	
Oral glucose	75 g OGTT
Tolerance tests	IADPSG
	WHO
	NICE
	100 g OGTT
	Carpenter-coustan
	NDDG
Fasting plasma glucose level	Based on WHO criteria (1)
[Absence of] pregestational diabetes mellitus criteria	See above

OGTT (Oral glucose tolerance test); IADPSG (International Association of Diabetes and Pregnancy Study Groups); WHO (World Health Organization); NICE (The National Institute for Health and Care Excellence, UK); NDDG (National Diabetes Data Group) (see Table 1).

**Table 1:** Diagnostic oral glucose tolerance tests based on organization or country guidelines.

Test	Guidelines	Number of abnormal values necessary for diagnosis	Fasting plasma glucose mmol/l (mg/dl)	1-h plasma glucose mmol/l (mg/dl)	2-h plasma glucose mmol/l (mg/dl)	3-h plasma glucose mmol/l (mg/dl)	Timing
75 g OGTT							
	WHO 2013 [1]	1	⩾5.1–6.9 (92–125)	⩾10.0 (180)	⩾8.5–11.0 (153–199)	N/A	24–28 wks
	IADPSG [25]	1	⩾5.1 (92)	⩾10.0 (180)	⩾8.5 (153)	N/A	
	NICE (UK) [26]	1	⩾5.6 (101)	Not required	⩾7.8 (140)	N/A	24–28 wks
100 g OGTT							
	Carpenter Coustan [27]	2	⩾5.3 (95)	⩾10.0 (180)	⩾8.6 (155)	⩾7.8 (140)	24–28 wks
	NDDG [27]	2	⩾5.8 (105)	⩾10.6 (190)	⩾9.2 (165)	⩾ 8.0 (145)	

OGTT (Oral glucose tolerance test); IADPSG (International Association of Diabetes and Pregnancy Study Groups); WHO (World Health Organization); NICE (The National Institute for Health and Care Excellence, UK); NDDG (National Diabetes Data Group).

Ideally, a postpartum or interpregnancy glucose tolerance test would be performed to confirm that the diagnosis of diabetes mellitus is confined to pregnancy and to exclude diabetes mellitus outside of pregnancy. Postpartum or interpregnancy GTTs, however, are infrequently performed and therefore the absence of this test would not be exclusionary.

## 3 Guidelines for data collection, analysis and presentation of gestational diabetes mellitus

It was the consensus of the Brighton Collaboration *Gestational Diabetes Mellitus Working Group* to recommend the following guidelines to enable meaningful and standardized collection, analysis, and presentation of information about gestational diabetes. However, implementation of all guidelines might not be possible in all settings. The availability of information may vary depending upon resources, geographical region, and whether the source of information is a prospective clinical trial, a post-marketing surveillance or epidemiological study, or an individual report of gestational diabetes. Also, as explained in more detail in the overview paper in this volume, these guidelines have been developed by this working group for guidance only, and are not to be considered a mandatory requirement for data collection, analysis, or presentation.

### 3.1 Data collection

These guidelines represent a desirable standard for the collection of data on availability following immunization to allow for comparability of data, and are recommended as an addition to data collected for the specific study question and setting. The guidelines are not intended to guide the primary reporting of GDM to a surveillance system or study monitor. Investigators developing a data collection tool based on these data collection guidelines also need to refer to the criteria in the case definition, which are not repeated in these guidelines.

Guidelines 1–44 below have been developed to address data elements for the collection of adverse event information as specified in general drug safety guidelines by the International Conference on Harmonization of Technical Requirements for Registration of Pharmaceuticals for Human Use [28], and the form for reporting of drug adverse events by the Council for International Organizations of Medical Sciences [29]. These data elements include an identifiable reporter and patient, one or more prior immunizations, and a detailed description of the adverse event, in this case, of GDM following immunization. The additional guidelines have been developed as guidance for the collection of additional information to allow for a more comprehensive understanding of GDM following immunization.

#### 3.1.1 Source of information/reporter

For all cases and/or all study participants, as appropriate, the following information should be recorded:

(1) Date of report.
(2) Name and contact information of person reporting (Footnote 2) and/or diagnosing the GDM as specified by country-specific data protection law.
(3) Name and contact information of the investigator responsible for the subject, as applicable.
(4) Relation to the patient (e.g., immunizer [clinician, nurse], family member [indicate relationship], other).

#### 3.1.2 Vaccinee/control

##### 3.1.2.1 Demographics

For all cases and/or all study participants, as appropriate, the following information should be recorded:

(5) Case/study participant identifiers (e.g. first name initial followed by last name initial with medical record number/booking number/subject number) or alpha-numeric code (or in accordance with country-specific data protection laws).
(6) Date of birth, age, and sex.
(7) For infants: Gestational age and birth weight and length, and whether multiple gestation. Infant’s name and identifier (medical record number/booking number/subject number or alpha-numeric code) should also be recorded.

##### 3.1.2.2 Clinical and immunization history

For all cases and/or all study participants, as appropriate, the following information should be recorded:

(8) For the purposes of this definition, any hyperglycemia or diabetes diagnosis prior to current pregnancy or between pregnancies.
(9) Past medical history, including hospitalizations, underlying diseases/disorders, pre-immunization signs and symptoms including identification of indicators for, or the absence of, a history of allergy to vaccines, vaccine components or medications; food allergy; allergic rhinitis; eczema; asthma. Risk factors for GDM including family history of diabetes, GDM in prior pregnancies, eating habits and physical activity, weight or body mass index (BMI) at first prenatal visit.
(10) Any medication history (other than treatment for the event described) prior to, during, and after immunization including prescription and non-prescription medication as well as medication or treatment with long half-life or long term effect. (e.g. immunoglobulins, blood transfusion and immunosuppressants).
(11) Immunization history (i.e. previous immunizations and any adverse event following immunization (AEFI)), in particular occurrence of GDM after a previous immunization.

#### 3.1.3 Details of the immunization

For all cases and/or all study participants, as appropriate, the following information should be recorded:

(12) Date and time of immunization(s).
(13) Description of vaccine(s) (name of vaccine, manufacturer, lot number, dose (e.g. 0.25 mL, 0.5 mL, etc.) and number of dose if part of a series of immunizations against the same disease).
(14) If applicable, description of diluent (manufacturer, lot number, amount (e.g. 0.25 mL, 0.5 mL, etc.)
(15) The anatomical sites (including left or right side) of all immunizations (e.g. vaccine A in proximal left lateral thigh, vaccine B in left deltoid).
(16) Route and method of administration (e.g. intramuscular, intradermal, subcutaneous, and needle-free (including type and size), other injection devices).
(17) Needle length and gauge.

#### 3.1.4 The adverse event

(18) For all cases at any level of diagnostic certainty and for reported events with insufficient evidence, the criteria fulfilled to meet the case definition should be recorded.

Specifically document:

(19) Clinical description of signs and symptoms of GDM, and if there was medical confirmation of the event (i.e. patient seen by qualified health professional).
(20) Date/time of onset,^4^ first observation^5^ and diagnosis,^6^ end of episode^7^ and final outcome.^8^
(21) Concurrent signs, symptoms, and diseases.
(22) Measurement/testing.
  • Values and units of routinely measured parameters (e.g. blood glucose levels including fasting and postprandial measurements, temperature, blood pressure) – in particular those indicating the severity of the event;
  • Method of measurement (e.g. serum sampling or fingerstick for capillary glucose measurements, type of thermometer, oral or other route, duration of measurement, etc.);
  • Results of laboratory examinations, surgical and/or pathological findings and diagnoses if present.
(23) Treatment given for GDM especially specify whether diet-controlled or whether hyperglycemic agents were used and dosing.
(24) Outcome (Footnote 7) at last observation.
(25) Objective clinical evidence supporting classification of the event as “serious”.^9^
(26) Exposures other than the immunization 24 h before and after immunization (e.g. food, environmental, pharmaceutical) considered potentially relevant to the reported event.

#### 3.1.5 Miscellaneous/general

(27) The duration of surveillance for GDM should be predefined based on
  • Biologic characteristics of the vaccine e.g. live attenuated versus inactivated component vaccines.
  • Biologic characteristics of the vaccine-targeted disease.
  • Biologic characteristics of GDM including patterns identified in previous trials (e.g. early-phase trials).
  • Biologic characteristics of the vaccinee (e.g. nutrition, underlying disease like immunosuppressing illness).
(28) The duration of follow-up reported during the surveillance period should be predefined likewise. It should aim to continue to resolution of the event.
(29) Methods of data collection should be consistent within and between study groups, if applicable.
(30) Follow-up of cases should attempt to verify and complete the information collected as outlined in data collection guidelines 1–25.
(31) Investigators of patients with GDM should provide guidance to reporters to optimize the quality and completeness of information provided.
(32) Reports of GDM should be collected throughout the study period regardless of the time elapsed between immunization and the adverse event. If this is not feasible due to the study design, the study periods during which safety data are being collected should be clearly defined.

### 3.2 Data analysis

The following guidelines represent a desirable standard for analysis of data on GDM to allow for comparability of data, and are recommended as an addition to data analyzed for the specific study question and setting.

(33) Reported events should be classified in one of the following five categories including the three levels of diagnostic certainty. Events that meet the case definition should be classified according to the levels of diagnostic certainty as specified in the case definition. Events that do not meet the case definition should be classified in the additional categories for analysis.

**Event classification in 5 categories**^10^

**Event meets case definition**

(1) Level 1: Criteria as specified in the GDM case definition
(2) Level 2: Criteria as specified in the GDM case definition
(3) Level 3: Criteria as specified in the GDM case definition

**Event does not meet case definition**

Additional categories for analysis

(4) Reported GDM with insufficient evidence to meet the case definition^11^
(5) Not a case of GDM^12^
(34) The interval between immunization and reported GDM could be defined as the date/time of immunization to the date/time of onset (Footnote 3) of the first abnormal glucose measurement. If few cases are reported, the concrete time course could be analyzed for each; for a large number of cases, data can be analyzed in the following increments:

Subjects with gestational diabetes mellitus by interval to presentation

**Table:** 

Interval^∗^	Number
<7 days after immunization	
7 – <14 days after immunization	
14 – <28 days after immunization	
28 – <42 days (6 weeks) after immunization	
Week increments thereafter	
Total

(35) The duration of a possible GDM could be analyzed as the interval between the date/time of onset (Footnote 2) of the first symptoms and/or signs consistent with the definition and the end of episode (Footnote 6) and/or final outcome (Footnote 7). Whatever start and ending are used, they should be used consistently within and across study groups.
(36) If more than one measurement of a particular criterion is taken and recorded, the value corresponding to the greatest magnitude of the adverse experience could be used as the basis for analysis. Analysis may also include other characteristics like qualitative patterns of criteria defining the event.
(37) The distribution of data (as numerator and denominator data) could be analyzed in predefined increments (e.g. measured values, times), where applicable. Increments specified above should be used. When only a small number of cases is presented, the respective values or time course can be presented individually.
(38) Data on GDM obtained from subjects receiving a vaccine should be compared with those obtained from an appropriately selected and documented control group(s) to assess background rates of hypersensitivity in non-exposed populations, and should be analyzed by study arm and dose where possible, e.g. in prospective clinical trials.

Ultimately, careful analysis of data is necessary given overlap between usual timing of vaccination in pregnancy and routine GDM screening between 24 and 28 weeks gestational age in order to avoid misleading conclusions regarding GDM and associations with vaccinations purely based on timing of vaccine administration and GDM diagnosis.

### 3.3 Data presentation

These guidelines represent a desirable standard for the presentation and publication of data on GDM following immunization to allow for comparability of data, and are recommended as an addition to data presented for the specific study question and setting. Additionally, it is recommended to refer to existing general guidelines for the presentation and publication of randomized controlled trials, systematic reviews, and meta-analyses of observational studies in epidemiology (e.g. statements of Consolidated Standards of Reporting Trials (CONSORT), of Improving the quality of reports of meta-analyses of randomized controlled trials (QUORUM), and of meta-analysis Of Observational Studies in Epidemiology (MOOSE), respectively) [30], [31], [32].

(39) All reported events of GDM should be presented according to the categories listed in guideline 32.
(40) Data on possible GDM events should be presented in accordance with data collection guidelines 1–25 and data analysis guidelines 32–37.
(41) Terms to describe GDM such as “well-controlled”, “poorly controlled”, “low-grade”, “mild”, “moderate”, “high”, “severe” or “significant” are highly subjective, prone to wide interpretation, and should be avoided, unless clearly defined.
(42) Data should be presented with numerator and denominator (n/N) (and not only in percentages), if available.

Although immunization safety surveillance systems denominator data are usually not readily available, attempts should be made to identify approximate denominators. The source of the denominator data should be reported and calculations of estimates be described (e.g. manufacturer data like total doses distributed, reporting through Ministry of Health, coverage/population based data, etc.).

(43) The incidence of cases in the study population should be presented and clearly identified as such in the text.
(44) If the distribution of data is skewed, median and range are usually the more appropriate statistical descriptors than a mean. However, the mean and standard deviation should also be provided.
(45) Any publication of data on GDM should include a detailed description of the methods used for data collection and analysis as possible. It is essential to specify:
  • The study design.
  • The method, frequency and duration of monitoring for GDM.
  • The trial profile, indicating participant flow during a study including drop-outs and withdrawals to indicate the size and nature of the respective groups under investigation.
  • The type of surveillance (e.g. passive or active surveillance).
  • The characteristics of the surveillance system (e.g. population served, mode of report solicitation).
  • The search strategy in surveillance databases.
  • Comparison group(s), if used for analysis.
  • The instrument of data collection (e.g. standardized questionnaire, diary card, report form).
  • Whether the day of immunization was considered “day one” or “day zero” in the analysis.
  • Whether the date of onset (Footnote 3) and/or the date of first observation (Footnote 4) and/or the date of diagnosis (Footnote 5) was used for analysis.
  • Use of this case definition for GDM, in the abstract or methods section of a publication.^13^

## Disclaimer

The findings, opinions and assertions contained in this consensus document are those of the individual scientific professional members of the working group. They do not necessarily represent the official positions of each participant’s organization (e.g., government, university, or corporation). Specifically, the findings and conclusions in this paper are those of the authors and do not necessarily represent the views of their respective institutions.

## Footnotes

3 If the reporting center is different from the vaccinating center, appropriate and timely communication of the adverse event should occur.

4 The date and/or time of onset is defined as the time post immunization, when the GDM is first detect via diagnostic criteria described above. GDM is usually screen detected and therefore asymptomatic. This may only be possible to determine in retrospect.

5 The date and/or time of first observation of the first sign or symptom indicative for GDM can be used if date/time of onset is not known.

6 The date of diagnosis of an episode is the day post immunization when the event met the case definition at any level.

7 The end of an episode is defined as the time the event no longer meets the case definition at the lowest level of the definition.

8 E.g. recovery to pre-immunization health status, spontaneous resolution, therapeutic intervention, persistence of the event, sequelae, death.

9 An AEFI is defined as serious by international standards if it meets one or more of the following criteria: (1) it results in death, (2) is life-threatening, (3) it requires inpatient hospitalization or results in prolongation of existing hospitalization, (4) results in persistent or significant disability/incapacity, (5) is a congenital anomaly/birth defect, (6) is a medically important event or reaction.

10 To determine the appropriate category, the user should first establish, whether a reported event meets the criteria for the lowest applicable level of diagnostic certainty, e.g. Level three. If the lowest applicable level of diagnostic certainty of the definition is met, and there is evidence that the criteria of the next higher level of diagnostic certainty are met, the event should be classified in the next category. This approach should be continued until the highest level of diagnostic certainty for a given event could be determined. Major criteria can be used to satisfy the requirement of minor criteria. If the lowest level of the case definition is not met, it should be ruled out that any of the higher levels of diagnostic certainty are met and the event should be classified in additional categories four or five.

11 If the evidence available for an event is insufficient because information is missing, such an event should be categorized as “Reported GDM with insufficient evidence to meet the case definition”.

12 An event does not meet the case definition if investigation reveals a negative finding of a necessary criterion (necessary condition) for diagnosis. Such an event should be rejected and classified as “Not a case of GDM”.

13 Use of this document should preferably be referenced by referring to the respective link on the Brighton Collaboration website (http://www.brightoncollaboration.org).
