# Supplementary material for: Gestational diabetes mellitus: Case definition & guidelines for data collection, analysis, and presentation of immunization safety data
Source: Vaccine. 2017 Dec 4;35(48Part A):6555–62. doi: 10.1016/j.vaccine.2017.01.043 (PMC5710985; doi:10.1016/j.vaccine.2017.01.043)
Supplement: Supplementary data 1 [file mmc1.docx]

**APPENDIX A: Case definition Preterm Birth (Quinn et al. 2016, pp.6051-6052)**

***Prematurity and Assessment of Gestational Age Criteria:***

***Definitions of terms used*:**

- **Intrauterine Insemination (IUI)** - A procedure in which a fine catheter is inserted through the cervix into the uterus to deposit a sperm sample directly into the uterus, to achieve fertilization and pregnancy.
- **Embryo Transfer-** the procedure in which one or more embryos are placed in the uterus or fallopian tube.
- **Ultrasound (U/S) [62]** - 1^st^ trimester (≤13 6/7 weeks)

- 2^nd^ trimester scan (14 0/7- 27 6/7 weeks)

*- 3^rd^ trimester (28 0/7 + weeks)*

- **LMP (Last Menstrual Period) -** Gestational age is calculated from the first day of the mother’s last menstrual period**.**

***If LMP and U/S do not correlate, default to U/S GA assessment***

***Certain LMP:** **(LMP date + 280 days):**

Use LMP if within 7 days at < 14 weeks; within 14 days at < 26 weeks; within 21 days beyond 26 weeks.

***Uncertain** **LMP**- **First Trimester (≤ 13 6/7 weeks by LMP):**

Use the approximate date of the last menstrual period (LMP) if corroborated by physical exam, or a first trimester ultrasound. If there is a discrepancy of > 7 days between the LMP and the first trimester ultrasound, the ultrasound-established dates will take preference over LMP for gestational age dating.

***Uncertain** **LMP**- **Second Trimester (14 0/7- 27 6/7 weeks by LMP)**:

Use the approximate date of the LMP if corroborated by physical exam including fundal height, or a second trimester ultrasound. If there is a discrepancy of >10 days between the LMP and the second trimester ultrasound, the ultrasound-established dates will take preference over LMP for gestational age dating.

***Uncertain** **LMP- Third Trimester > 28 weeks**- third trimester ultrasound.

***No LMP date**:

If menstrual dates are unknown, the ultrasound-established dates will be usedfor gestational age dating or 2^nd^ trimester fundal height and/or newborn physical examination

- **Pregnancy symptoms** - nausea, fatigue, tender swollen breasts, frequent urination.
- **Antenatal Physical Examination** - pelvic bimanual examination confirming enlarged uterus [63]
- **Newborn Physical Examination - New Ballard Score**- physical and neurological assessment ***- Appendix 1***
- **Fundal Height (FH) in cms *- Appendix 2***
- **Birth Weight (BW) in grams - *Appendix 2***

***Prematurity and Assessment of Gestational Age:***

***Level 1: (highest level of certainty):***

**1**. **Certain LMP * or Intrauterine insemination (IUI) date or Embryo Transfer (ET) date *with* confirmatory 1^st^ trimester scan (≤ 13 6/7 weeks)**

***OR***

**2.** **1^st^ trimester scan (≤ 13 6/7 weeks)**

***Level 2A:***

**1**. **Certain LMP* *with* 2^nd^ trimester scan (14 0/7 weeks to 27 6/7 weeks)***. If LMP and U/S do not correlate, default to U/S GA assessment.*

***OR***

**2**. **Certain LMP* *with* 1^st^ trimester physical examination**

***Level 2B:***

**Uncertain LMP with 2^nd^ trimester scan (14 0/7 weeks to 27 6/7 weeks)**

***Level 3A:***

1. **Certain LMP *with* 3^rd^ trimester scan- 28 0/7 weeks +**

***OR***

**2**. **Certain LMP *with* confirmatory 2^nd^ trimester FH**

***OR***

**3. Certain LMP with birth weight**

***OR***

**4. Uncertain LMP with 1^st^ trimester physical examination**

***Level 3B:***

1. **Uncertain LMP *with* FH.**

***OR***

**2.** **Uncertain LMP *with* newborn physical assessment.**

***OR***

**3.** **Uncertain LMP *with* Birth weight**

Reference:

Quinn J, Munoz FM, Gonik B, Frau L, Cutland C, Mallett-Moore T, Kissou A, Wittke F, Das M, Nunes T, Pye S, Watson W, Alguacil Ramos A, Cordero JF, Huang W, Kochhar S, Buttery J, The Brighton Collaboration Preterm Birth Working Group (2016) Preterm birth: Case definition &amp; guidelines for data collection, analysis, and presentation of immunisation safety data. Vaccine 34(49): 6047-6056.
